# The Diagnostic Performance of a Four-Gene Digital Droplet PCR Panel for Urine Liquid Biopsy in Urothelial Bladder Cancer

**DOI:** 10.3390/diagnostics16010069

**Published:** 2025-12-24

**Authors:** Mark Jain, Alexander Tivtikyan, Dmitry Kislyakov, Tagir Rakhmatullin, David Kamalov, Vladislav Kokarev, Lolita Vorobeva, Larisa Samokhodskaya, Maria Zvereva, Armais Kamalov

**Affiliations:** 1 University Clinic, Lomonosov Moscow State University, 119992 Moscow, Russia; aleksandertivtikyan@yandex.ru (A.T.); dakislyakov@gmail.com (D.K.); tagir.rakhmatullin@internet.ru (T.R.); davidffm@gmail.com (D.K.); slm61@mail.ru (L.S.); priemnaya@mc.msu.ru (A.K.); 2 Faculty of Fundamental Medicine, Lomonosov Moscow State University, 119992 Moscow, Russia; kokarev.vladislav01@bk.ru (V.K.); v.lolita04@yandex.ru (L.V.); 3 Faculty of Chemistry, Lomonosov Moscow State University, 119991 Moscow, Russia; mzvereva@chem.msu.ru

**Keywords:** liquid biopsy, urothelial cancer, bladder cancer, cell-free DNA, mutations, urine, *TERT*, *GPR126*, *FGFR3*, *PIK3CA*

## Abstract

**Background**: Urothelial bladder carcinoma (UBC) is a disease that lacks robust non-invasive laboratory biomarkers. Recently, urine liquid biopsy has emerged as a promising tool for diagnosis and surveillance of patients with these tumors. The aim of this study was to evaluate the diagnostic potential of a urinary tumor DNA detection panel, which included eight common point mutations in *TERT*, *GPR126*, *FGFR3*, and *PIK3CA* genes, in UBC. **Methods**: The study included patients with histologically confirmed UBC (*n* = 88) and patients with cystitis, bladder leiomyomas, or other non-malignant conditions (control group; *n* = 72). DNA was extracted from whole urine specimens. ddPCR analysis was performed using the Bio-Rad QX200 AutoDG ddPCR system. **Results**: Urinary tumor DNA detection panel demonstrated a sensitivity of 78.4% and a specificity of 100% (AUC−ROC = 0.892). Detection rates for the analyzed mutations were the following: 54.5%, 37.5%, 28.4%, and 38.6% for *TERT*, *GPR126*, *FGFR3*, and *PIK3CA*, respectively. Pairwise comparisons of mutant allele fractions (MAFs) for samples simultaneously positive for ≥2 mutations revealed an absence of significant differences (*p* > 0.05), except for the pair of *FGFR3* vs. *PIK3CA* (*p* = 0.03). MAFs were not associated with any clinical and demographic features (*p* > 0.05), with the only exception being the tumor size: patients with tumors larger than 2.16 cm^3^ had higher MAFs than the rest (23.4 [1.8; 46.3] vs. 1.6 [0; 24.6] %, respectively, *p* = 0.02). **Conclusions**: Upon further validation, the presented tumor DNA detection panel for ddPCR might become a useful tool for diagnostic purposes in UBC.

## 1. Introduction

Bladder cancer (BC) is the most frequent oncological disease of the urinary tract [1]. Recent Global Cancer Statistics report revealed that this cancer ranks 9th in incidence and 13th in mortality worldwide [2]. It is projected that by 2040 that annual BC cases and deaths will increase by 73% and 87%, respectively, highlighting the urgent need to develop new diagnostic and treatment approaches to tackle its growing global burden [3]. This oncological disease is approximately four times more frequent in men, which is largely explained by its main risk factors: tobacco smoking and occupational exposure to aromatic amines and hydrocarbons (dye and rubber industry) [4]. Additionally, emerging evidence suggests that several dietary features, microbiome imbalance, diesel exhaust exposure, pelvic radiotherapy, and specific gene-environment interactions may contribute to the development of BC [1].

There are several types of BC, which are classified based on the type of cells where cancer originated. Urothelial bladder carcinoma (UBC) is the most common (80–95%) type that starts from the bladder lining’s urothelial cells; it is subclassified into muscle-invasive and non-muscle-invasive subtypes, with the latter being 2–3 times more frequent and less lethal [5]. Other types include squamous cell carcinoma (originates in flat cells), adenocarcinoma (originates in glandular cells), with sarcomatoid carcinoma (originates in muscle cells and in some other) and small-cell carcinoma (originates in neuroendocrine cells) being the rarest [6,7]. It is worth noting that these types of BC are characterized by a drastically different genetic landscape [8,9]. The diagnostic panel, which is described in the present study, was tailored to the mutational profile of urothelial carcinoma and tested in the UBC population. Thus, it will not perform equally in other types of BC.

UBC is known for its exceptionally high recurrence rate. According to a recent multicenter retrospective observational study, its 1-, 2-, and 5-year recurrence-free survival rates were 81.6%, 72.4%, and 59.2%, respectively, whereas the overall rate of recurrence with progression was 6.2% [10]. Therefore, following cancer resection, patients require active surveillance for up to 5 years (every 3–6 months), making the management of this disease extremely expensive for healthcare providers [11,12].

Currently, cystoscopy and urine cytology are the main diagnostic tools widely used in clinical practice for UBC. Cystoscopy is effective at any tumor stage, but it has several major drawbacks, including high cost, the need of sedation/anesthesia, and high inter- and intraobserver variability [13,14]. Moreover, due to its invasive nature, patients may experience various adverse events, including urinary tract infections, bladder cramping, hematuria, frequent urination, decline in libido, and discomfort during urination [15,16]. Urine cytology is a non-invasive technique, which is characterized by overall sensitivity of 37–67% and specificity of 89–95% (depending on the tumor grade and stage) [17,18]. It is also prone to the effect of inter- and intraobserver variability, and its results may be affected by several factors, such as infections and urolithiasis [19,20].

Tumor DNA (tDNA) analysis-based liquid biopsy has emerged as a promising diagnostic tool in BC, which is largely devoid of the above-mentioned shortcomings [21]. Urine is a great source of tDNA, as it resides in a state of prolonged contact with the surface of the tumor. Thus, this biomaterial may contain large amounts of tDNA from exfoliated malignant cells, apoptosis-related cell-free tDNA, as well as exosomal tDNA [22]. There are several approaches to distinguish tDNA from the DNA of normal cells, including methylation profiling, fragmentation analysis, and mutation detection [23]. The latter is particularly useful in UBC, as this disease is characterized by a large number of common point mutations [9]. Moreover, the frequency of mutations in hotspots of several genes (*TERT*, *GPR126*, *FGFR3*, and *PIK3CA*) is so high (up to 90% combined [8]) that it enables the use of cheaper targeted analytical approaches, such as digital droplet polymerase chain reaction (ddPCR) and its analogs, instead of sequencing techniques.

The aim of this study was to evaluate the diagnostic potential of a urinary tDNA detection panel for ddPCR, which included eight common point mutations in *TERT*, *GPR126*, *FGFR3*, and *PIK3CA* genes in UBC.

## 2. Materials and Methods

### 2.1. General Information

Patient enrollment took place from April 2020 to October 2024. Informed consent was obtained from each study participant. Overall, the study included 160 individuals. A total of 88 patients had histologically confirmed urothelial carcinoma (UBC group; predominantly at early stages), 72 patients had cystitis, bladder leiomyomas, or other non-malignant conditions (control group). Dimensional characteristics of the tumors were evaluated using transabdominal 3D ultrasonography on a SonoAce X8 instrument (Samsung Medison Co., Ltd., Seoul, Republic of Korea). The demographic and clinical characteristics of study participants are presented in Table 1, whereas detailed depersonalized data is available in Table S1.

### 2.2. Biomaterial Collection and Sample Preparation

An aliquot of first morning urine (50–100 mL) was obtained from each study participant. In both groups, biomaterial was collected prior to any invasive procedures. Urine samples were stored at −80 °C. DNA was isolated from intact urine samples (4 mL) using a QIAamp Circulating Nucleic Acid kit (Qiagen GmbH, Hilden, Germany) according to the manufacturer’s instructions with a slight adjustment of the protocol. The lysis step was extended for additional 20 min to ensure complete digestion of any cell particles in the biomaterial. It is worth noting that the manufacturer’s protocol for the above-mentioned DNA extraction kit was designed for use with urine supernatant. In our experience, the extension of the lysis step was essential to prevent the column plugging at binding and wash steps of the DNA extraction process. The DNA elution volume was 100 μL.

As the extracted DNA solutions contained an abundance of carrier RNA, the spectrophotometric assessment of their quality was not informative. Therefore, sample quality was ensured using real-time PCR as described below.

### 2.3. Urinary DNA Analysis

In this study, ddPCR was the analytical method of choice. It was carried out using the QX200 AutoDG ddPCR System (Bio-Rad Laboratories, Inc., Hercules, CA, USA). DNA amplification was performed on the Veriti Thermal Cycler (ThermoFisher Scientific, Inc., Waltham, MA, USA). Sample quality assessment via real-time PCR was carried out using the CFX96 instrument System (Bio-Rad Laboratories, Inc., Hercules, CA, USA).

The urinary tDNA detection panel included 8 non-coding and coding genetic alterations: *TERT* promoter (*pTERT*) mutations at two positions upstream of the transcription starting site (at −124 bp, chr. 5: 1,295,228, G/A substitution; and −146 bp, chr. 5: 1,295,250, G/A substitution, genome assembly GRCh37, in the literature these are also known as C228T and C250T [24]); *GPR126* 6th intron mutations (chr. 6: 142,706,206, G/A substitution, and chr. 6: 142,706,209, C/T substitution, these are also known to be present simultaneously in some cases); *FGFR3* mutation S249C; and *PIK3CA* mutations E542K, E545R, H1047R). *pTERT* and *GPR126* 6th intron mutations were analyzed using two different previously described screening assays [25,26]. These assays do not allow for discrimination of mutations in the corresponding genes (both mutant allele detection probes are labeled with the same fluorophore). Therefore, it was not possible to differentiate *pTERT* C228T from C250T mutations; the same was true for *GPR126* 6th intron mutations. Singleplex assays were used for the analysis of *FGFR3* S249C, *PIK3CA* E542K, *PIK3CA* E545K, *PIK3CA* H1047R mutations. A detailed description (including oligonucleotide sequences, thermocycling conditions and recommended concentrations in the final ddPCR mixture) of all assays included in the urinary tDNA detection panel is available in Table S2. The selection of mutations for the urinary tDNA detection panel was based on the following criteria:

- High frequency in UBC [8,27,28,29,30].
- Low degree of genotype overlaps. For example, mutations in *LEPROTL1* are more frequent than the selected alterations in *FGFR3* and *PIK3CA*. However, they are often simultaneously present with mutations in *GPR126*, providing no benefit for the diagnostic sensitivity [30].
- Convenience of assay development. There are several frequent non-coding mutations in UBC [30], yet the exceptionally high GC-content in those DNA regions impedes the development of assays for ddPCR.

These assays were preclinically validated using either DNA samples with known positive mutational status, or synthetic DNA constructs carrying respective mutations as described previously [26,31]. Cutoff values for false-positive droplets were established for each assay in a series of experiments with different inputs of wild-type DNA. For *pTERT*, *FGFR3* and *PIK3CA* assays, the cutoff values were set to 2 false-positive droplets per reaction well. In the case of *GPR126* assay, the number of false-positive droplets positively correlated with the wild-type DNA input. Therefore, for this assay the cutoff value was set to 1.85% of mutant allele fraction (MAF). The above-mentioned cutoff values were subtracted from the results of the tDNA analysis for each respective assay to ease the interpretation of the quantitative data.

The quality control of the extracted DNA samples was carried out using a 5X qPCRmix-HS SYBR (Evrogen, JSC, Moscow, Russia) and primers from the *GPR126* assay (Table S2) (the criterion was a threshold cycle of less than 32). Due to the pronounced heterogeneity of the samples, normalization of DNA concentration was not implemented. Urinary DNA samples were analyzed in a single measurement. Reactions were repeated for wells that did not generate at least 10,000 droplets. The mean number of accepted droplets during the reading step of ddPCR was 20,347 [95% confidence interval (CI): 20,173–20,521]. Each ddPCR plate had negative and positive controls for the respective tDNA detection assays.

Results of tDNA analysis are presented as MAFs using the following formula:

(1) MAF = C_mutated DNA_/(C_wild type DNA_ + C_mutated DNA_) × 100%,

where “C” is the DNA concentration in copies/μL of the ddPCR mixture. If a sample was simultaneously positive for 2 or more mutations, MAF was presented for a mutation with its highest value. For the sake of convenience, all copies of mutated DNA found in whole urine specimens are referred to as “tDNA”, although we acknowledge that these molecules might originate from other sources (for example, from apoptotic senescent cells).

Data for *GPR126* 6th intron mutations analysis for 70 UBC patients included in this study was extracted from our previous report, which was dedicated to the development of a respective screening assay [26].

### 2.4. Statistical Analysis

IBM SPSS Statistics 26.0 Software (IBM Corp., Armonk, NY, USA) was utilized for the data processing. The normality of data distribution was assessed using Shapiro–Wilk’s test. Since all variables included in the analysis did not have a normal distribution, nonparametric statistical methods were used. Paired variables were compared using the Wilcoxon test, whereas unpaired variables—using the Mann–Whitney U test. Correlation was assessed by means of Spearman’s rank coefficient (r_s_). The associations between variables were evaluated using Fisher’s exact test. Continuous data are presented as median [quartile 1; quartile 3]. Receiver operating characteristic (ROC) analysis was performed to evaluate the diagnostic potential of the urinary tDNA detection panel. *p* < 0.05 was considered statistically significant.

## 3. Results

**Figure 1:**
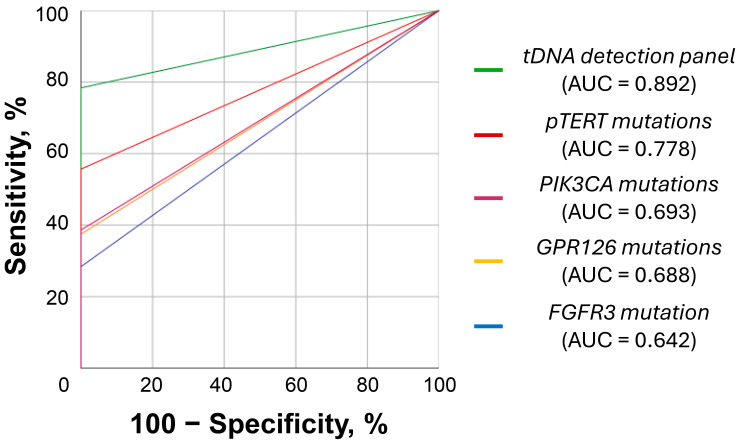
ROC curves for the developed urinary tDNA detection panel. *pTERT* mutations included substitutions in two positions upstream of the transcription starting site (at −124 bp, chr. 5: 1,295,228, G/A substitution; and −146 bp, chr. 5: 1,295,250, G/A substitution, genome assembly GRCh37). *PIK3CA* mutations included 3 common substitutions in the coding regions of the gene (E545K, E542K, H1047R). *GPR126* mutations included two substitutions in its 6th intron (chr. 6: 142,706,206, G/A substitution, and chr. 6: 142,706,209, C/T substitution, genome assembly GRCh37). For *FGFR3* only 1 coding mutation was analyzed, namely S249C. ROC, receiver operating characteristic; *pTERT*, promoter of the *TERT* gene; AUC, area under the ROC curve.

Urinary DNA was successfully analyzed in all samples from 160 individuals included in the study. Detailed results for each sample are available in Table S1. ROC-curves illustrating the diagnostic potential of the urinary tDNA detection panel are presented in Figure 1. tDNA carrying all analyzed mutations was absent in the control group’s urine. Thus, the specificity of the tDNA detection panel was 100% [95% CI: 100–100%], whereas the sensitivity was 78.4% [95% CI: 69.8–87.0%] (tDNA was detected in 69/88 UBC patients). The resulting AUC for the panel was 0.892 [95% CI: 0.839–0.946], highlighting its satisfactory diagnostic performance. It is worth noting that the control group had lower total urinary DNA levels than the UBC group, although the difference did not reach statistical significance (3242.7 [1395.0; 14220.5] vs. 8399.4 [1606.7; 38684.3] copies/mL, respectively, *p* = 0.073; Figure S1a). As a diagnostic biomarker for UBC, total DNA level performed poorly (AUC of 0.598 [95% CI: 0.498–0.697]; Figure S1b). *pTERT* mutations were the most frequent (54.5%), followed by *PIK3CA* and *GPR126* mutations (detection rates of 38.6% and 37.5%, respectively). *FGFR3* gene was mutated most rarely (28.4%). Within the analyzed *PIK3CA* mutations, detection rates were the following: 23.9%, 17.0%, and 9.1% for E545K, E542K, and H1047R mutations, respectively.

**Figure 2:**
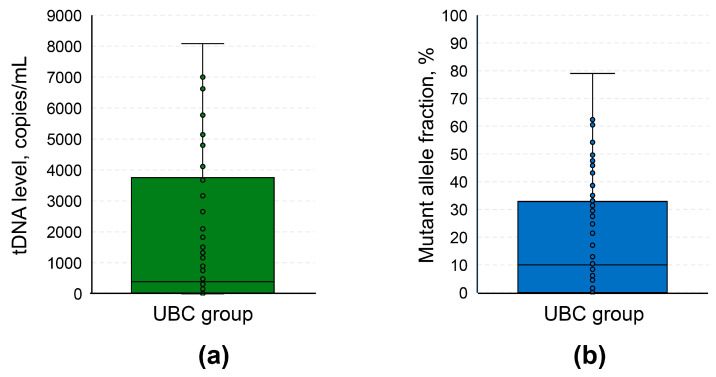
Boxplots for the distribution of tDNA analysis results in the UBC group. (**a**) tDNA level is expressed in copies per 1 mL of urine. (**b**) Mutant allele fraction is expressed in %. If a sample was simultaneously positive for 2 or more mutations, tDNA level and mutant allele fraction were selected for a mutation with the highest value of the corresponding variable. Several outliers with tDNA levels exceeding 9000 copies/mL were excluded to maintain the proportions of the plot. UBC, urothelial bladder cancer; tDNA, tumor DNA.

**Figure 3:**
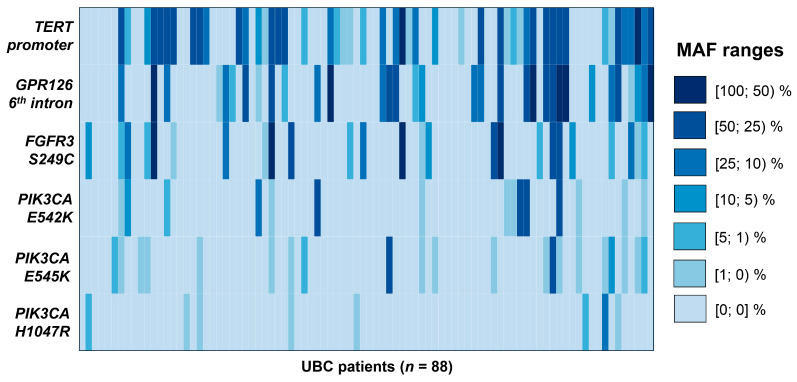
Heatmap for the analysis of mutations included in the urinary tDNA detection panel. *TERT* promoter mutations included substitutions in two positions upstream of the transcription starting site (at −124 bp, chr. 5: 1,295,228, G/A substitution; and −146 bp, chr. 5: 1,295,250, G/A substitution, genome assembly GRCh37). *GPR126* mutations included two substitutions in its 6th intron (chr. 6: 142,706,206, G/A substitution, and chr. 6: 142,706,209, C/T substitution, genome assembly GRCh37). The heatmap scale was segmented to ease the interpretation of values in samples with low MAFs. MAF, mutant allele fraction; UBC, urothelial bladder cancer.

In the UBC group, tDNA levels in the urine varied significantly: 385.7 [23.8; 3700.1] copies/mL (Figure 2a). The same degree of variance was observed for MAFs: 10.0 [0.05; 32.4] % (Figure 2b). The distribution of MAFs for each studied mutation per urinary DNA sample is available in the heatmap (Figure 3). Simultaneous presence of mutations included in the urinary tDNA detection panel was quite frequent, and more than half of patients in the UBC group had at least 2 mutations present in their samples.

**Figure 4:**
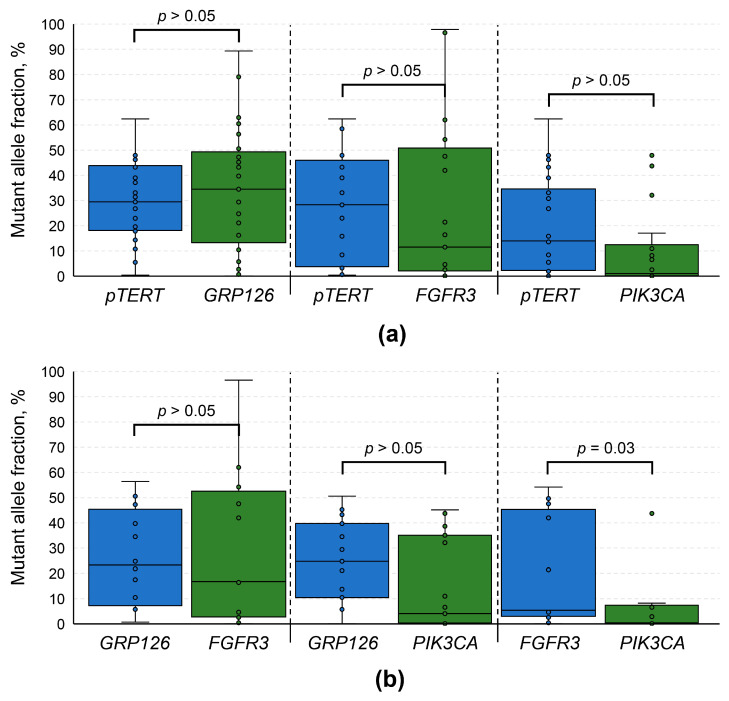
Pairwise comparisons of mutant allele fractions among samples simultaneously positive for at least two mutations included in the urinary tDNA detection panel. (**a**) Comparisons for *pTERT* vs. other genes. (**b**) Comparisons among *GPR126*, *FGFR3*, and *PIK3CA* genes pairs. *pTERT* mutations included substitutions in two positions upstream of the transcription starting site (at −124 bp, chr. 5: 1,295,228, G/A substitution; and −146 bp, chr. 5: 1,295,250, G/A substitution, genome assembly GRCh37). *PIK3CA* mutations included 3 common substitutions in the coding regions of the gene (E545K, E542K, H1047R). *GPR126* mutations included two substitutions in its 6th intron (chr. 6: 142,706,206, G/A substitution, and chr. 6: 142,706,209, C/T substitution, genome assembly GRCh37). For *FGFR3* only 1 coding mutation was analyzed, namely S249C. *pTERT*, promoter of the *TERT* gene.

**Figure 5:**
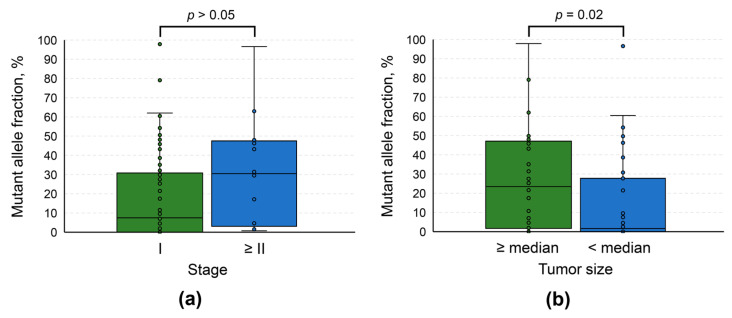
Box plots for comparisons of mutant allele fractions based on characteristics of the tumors. (**a**) Stages. (**b**) Tumor sizes. Median tumor size was 2.16 cm^3^. If a sample was simultaneously positive for 2 or more mutations, the mutant allele fraction was selected for a mutation with the highest value of the corresponding variable.

To obtain an insight into the genetic evolution of the tumors, we performed a set of pairwise comparisons of MAFs in urinary DNA samples simultaneously containing at least 2 of the studied mutations (analysis was performed for each pair of genes as presented in Figure 4). It appeared that in most cases MAFs did not differ significantly (*p* > 0.05), with the only exception being *FGFR3* vs. *PIK3CA*. Samples simultaneously positive for *FGFR3* and *PIK3CA* mutations were characterized by a slightly higher MAF for the former (*p* = 0.03), although the sample size for this comparison was quite small (*n* = 13). Overall, it might be assumed that these mutations occurred relatively at the same timepoint of tumorigenesis. We hypothesize that it was an early event in the process, as MAFs in patients with tumor stages I vs. II and above did not differ significantly (*p* > 0.05, Figure 5a). Moreover, comparison of detection rates for mutations included in the urinary tDNA detection panel did not reveal any statistically significant differences (*p* > 0.05, Figure S2).

Finally, we conducted an analysis of the associations of tDNA quantification results with various clinical and demographic characteristics of patients in the UBC group. Analysis revealed that patients with tumors larger than 2.16 cm^3^ (which was the median in our cohort) had higher MAFs than the rest (23.4 [1.8; 46.3] vs. 1.6 [0; 24.6] %, respectively, *p* = 0.02, Figure 5b). However, MAF was not associated with any other investigated qualitative parameters, including sex, tumor grade, history of smoking, and presence of macrohematuria (*p* > 0.05, Figure S3). There was no correlation of this variable with age as well (*p* > 0.05, Figure S4).

## 4. Discussion

Our urinary tDNA detection panel for ddPCR achieved a satisfactory diagnostic performance with a sensitivity and specificity of 78.4% and 100%, respectively. According to publicly available UBC sequencing datasets, the expected combined frequency of the mutations included in the panel was up to 90% [8,9,29]. Obviously, urine as a non-invasive source of tDNA has certain limitations, mainly regarding the overall concentration of DNA. In our cohort, most patients had less than 10,000 copies of genome per 1 mL of urine. In cases with MAFs at the lower end of the spectrum (below 0.5%), false-negative results were quite likely to occur, which might explain the discrepancy between the actual and expected tDNA detection rates.

Such high specificity was to some extent expected. Mutations in *pTERT*, *GPR126*, *FGFR3*, and *PIK3CA* are cancer-specific and are unlikely to occur in normal cells [32]. Moreover, none of them are linked to the clonal hematopoiesis of indeterminate potential (CHIP), a common condition in elderly individuals [33]. CHIP causes blood stem cells to acquire certain mutations (with mosaic loss of Y chromosome being the most frequent [34]) and thus generate multiple differentiated cells, which are released into the bloodstream [35]. It is believed that CHIP is responsible for the identification of mutated circulating DNA in seemingly cancer-free donors [36]. This DNA could pass the kidney’s glomerular filtration system (especially fragments less than 150 bp); hence, it might be present in the urine [37]. In this study, we deliberately chose to process and analyze urine samples from the study participants in mixed batches. On one hand, it could cause the splashome effect (contamination of neighboring wells by tDNA from samples with its high abundance) [38]. On the other, it replicated the possible conditions of the analysis in clinical practice. Therefore, the specificity of our urinary tDNA detection panel might drop upon the extension of the control group, but we do not expect the decrease to be significant.

In our cohort, detection rates of individual mutations in urinary DNA were mostly concordant with the tissue detection rates in the available literature. It is known that *pTERT* mutations are found in 59.1–77.0% of patients with urothelial carcinoma, compared to 54.5% in our study [27,28,39]. There is less data on the frequency of *GPR126* mutations, given their recent discovery, but the expected detection rate for these alterations is in the range of 31.4–53.0% (37.5% in our cohort) [29,30,39]. The frequency of *FGFR3* S249C mutations varies significantly (6.4–55.0% vs. 28.4% in our cohort), as this mutation is known to be dependent on the genetic background of the individual and the stage of the disease (*FGFR3* mutations are rare in some ethnic groups and in patients with an advanced disease) [40,41,42,43]. Detection rates for *PIK3CA* mutations are also reported in a wide range of values (10.0–38.0% vs. 38.6% in our cohort), and these mutations are associated with lower-stage tumors as well [44,45,46,47]. It is worth noting that our panel included three most common mutations in this gene, but not the entire spectrum of possible alterations. Therefore, the detection rate in our study was overall slightly higher than expected. It is known that the prevalence of *PIK3CA* mutations is associated with race, which might explain the discrepancy in the reported values [48]. In this study, *PIK3CA* mutations were found exclusively only in 6.8% of patients, thus the impact of the possible overdetection was minor.

Comparison of our findings to the results of other studies dedicated to the analysis of tDNA in urine is quite challenging due to several major methodological differences. Most studies focused on the analysis of either cell-free tDNA in urine supernatant, or tDNA in urine sediment, whereas we analyzed whole urine [24,39,49,50]. In our experience, whole urine provides a significantly higher DNA yield (up to 10 times more than sediment and up to 20 times more than supernatant), especially in UBC patients who often have a pronounced cellular component in their urine (Figure S5). Moreover, Z. Ou et al. demonstrated that the frequency of several mutations may drastically differ between the urine sediment and supernatant of the same individual [49]. DNA analysis methods may also significantly influence the results. Our study utilized ddPCR due to its resilience to PCR inhibitors, robustness in the low range of MAFs, and cheaper price compared to sequencing-based approaches [51,52,53,54]. The main disadvantage of this method is the inability to simultaneously analyze a large number of targets, which is why our urinary tDNA detection panel did not include other common genetic alterations associated with UBC (such as mutations in *TP53*, *ERBB2*, etc.). Other studies dedicated to the analysis of urinary tDNA employed a broad range of analytical techniques from quantitative real-time PCR to next-generation sequencing [55,56,57]. Studied cohorts were often small and included upper tract urothelial carcinoma, which is known to be genetically distinct from UBC [58]. Finally, to our knowledge, there is not a single study focused on analyzing the same combination of mutations in urinary DNA as it was in our study. Nevertheless, as far as a reliable comparison is possible, detection rates in our cohort are in line with those in other studies. Reported detection rates of urinary DNA carrying the studied mutations are the following: *pTERT* (28.6–94.0%), *GPR126* 6th intron (11.0–83.3%), *FGFR3* S249C (16.1–38.0%), and *PIK3CA* E545K, E542K, H1047R combined (13.0–28.2%) [39,49,50,55,59,60].

Even though the presented urinary tDNA detection panel achieved quite a high sensitivity of 78.4% in UBC patients, it may still need several modifications to push the tDNA detection rate even further. Assays for *PIK3CA* mutations could be merged to form a single-well screening assay (as was performed for *pTERT* and *GPR126* mutations). The same could be performed for *FGFR3* mutations (upon addition of several other frequent alterations in this gene). Reduction in the number of analyzed ddPCR wells per sample may open opportunities to add new targets to the panel, such as other frequent mutations or even different tDNA biomarkers. Recent studies suggest that the analysis of methylation and fragmentation patterns in urinary DNA might be a useful approach for non-invasive diagnosis of UBC [61,62,63]. Moreover, it was demonstrated that such underexplored genetic alterations as copy number variations could be efficiently detected using ddPCR in cell-free DNA of cancer patients [64]. They might become promising candidates for urinary tDNA detection panels, as it is known that UBC is characterized by several frequent copy number variations, including Y chromosome loss in males, *FGFR3* copy number gain and others [8,65].

According to the results of pairwise comparisons for urinary DNA samples with a simultaneous presence of two or more mutations and the absence of any significant associations of MAFs with tumor stage and grade, it might be hypothesized that the analyzed mutations were an early genetic event in tumorigenesis. This assumption might be supported by emerging evidence, which suggests that mutations mediated by Apolipoprotein B mRNA-editing enzyme catalytic polypeptide-like (APOBEC) may play a central causative role in driving UBC development and progression [66]. Recent data indicates that mutations in *pTERT*, *GPR126*, *FGFR3,* and *PIK3CA* genes are linked to the APOBEC dysregulation, confirming the claim above [56,67]. Taken together, these findings highlight that the presented urinary tDNA detection panel might be effective at the earliest stages of UBC and that it might be worth investigating its prognostic potential regarding the disease recurrence in postresection urine samples.

This study had certain limitations:

- The sample size was quite limited, which could have influenced the significance of our results. However, it is worth noting that all comparisons and ROC analyses presented herein had sufficient statistical power at thresholds of α = 0.05 and β = 0.20.
- The control group had a lower male-to-female ratio, fewer patients with smoking history and was slightly younger. We did not find any evidence of significant sex-specific differences for the detection rates of the studied mutations in the literature. As was mentioned previously, these mutations are not related to CHIP, thus we do not expect that a minor age difference could have significantly influenced the results.
- Several aspects of sample processing and analysis may have negatively impacted the detectability of tDNA, namely the use of frozen whole urine, low input volume of the biomaterial, and absence of technical replicates for ddPCR.
- This study did not include a comparison of mutational profiles in urine and tissue, as it was dedicated to the evaluation of diagnostic performance of the tDNA detection panel and not to the determination of its genotyping ability (from the clinical perspective, we do not find it necessary to non-invasively genotype early-stage UBC prior to the transurethral resection of the tumor). A false-positive rate of 0% in the control group at the preclinically defined cutoff values for each assay at least to some extent advocates for the absence or low level of mutation overdetection in the UBC group. However, we acknowledge that comparison of our data to tissue genotyping results might have strengthened the significance of our findings.
- This study did not include the assessment of the diagnostic performance of our urinary tDNA detection panel in patients stratified by molecular subtypes of the tumor. Even though *PIK3CA* mutations are present equally frequently across all molecular subtypes of BC, *FGFR3* and *pTERT* mutations are known to be associated with luminal papillary subtype (data for *GPR126* non-coding mutations in the literature is quite scarce) [68]. Thus, it is likely that the diagnostic performance of the presented panel may change in a cohort with a different structure of BC molecular subtypes.
- Our urinary tDNA detection panel lacks external validation, and this study did not include a separate validation set, therefore conclusions regarding its clinical utility might be drawn only after further investigation.

## 5. Conclusions

Urine liquid biopsy with tDNA identification is a promising diagnostic tool in UBC patients. Its non-invasive nature makes it feasible for routine implementation, especially in the postresection period for patients requiring constant follow-up. A reduction in the number of cystoscopies for recurrence monitoring is among the main goals of the liquid biopsy assay development. Our urinary tDNA detection panel for ddPCR demonstrated a satisfactory diagnostic performance; upon further validation it might become valuable for the management of patients with this disease. This panel was presented in a ready-to-use format, and we hope that it might be useful for other researchers aiming to analyze these mutations in UBC and beyond.

## Figures and Tables

**Table 1:** Demographic and clinical characteristics of study participants.

Parameters	UBC Group (*n* = 88)	Control Group (*n* = 72)
Age, years ^1^	63.8 (26.0–87.0)	51.0 (18.0–79.0)
Sex, n (%):		
-Male	74 (84.1%)	46 (63.9%)
-Female	14 (15.9%)	26 (36.1%)
History of smoking, n (%)	31 (35.2%)	6 (8.3%)
Macrohematuria, n (%)	12 (13.6%)	0 (0.0%)
Tumor stage, n (%):		
-I	75 (85.2%)	N/A
-II	5 (5.8%)	N/A
-IIIA	6 (6.8%)	N/A
-IIIB	1 (1.1%)	N/A
-IVA	1 (1.1%)	N/A
Tumor grade, n (%):		
-Low	42 (47.7%)	N/A
-High	21 (23.9%)	N/A
-Unknown	25 (28.4%)	N/A
Tumor size, cm^3 2^	2.16 [1.04–11.05]	N/A
Presence of metastases, n (%)	1 (1.1%)	N/A

UBC, urothelial bladder cancer; N/A, not applicable; ^1^ data presented as mean (range); ^2^ data presented as median [interquartile range].

## Data Availability

All data generated in the present study are available in Table S1.

## Supplementary Materials

The following supporting information can be downloaded at https://www.mdpi.com/article/10.3390/diagnostics16010069/s1, Figure S1: Comparison of total DNA levels on UBC and control groups; Figure S2: Detection rates of the analyzed mutations in relation to BC stage; Figure S3: Mutant allele fractions in relation to various clinical and demographic parameters; Figure S4: Scatter plot for age and mutant allele fraction; Figure S5: Comparison of total DNA levels for frozen biomaterial; Table S1: Detailed demographic, clinical, and analytical data for study participants; Table S2: Detailed description of assays included in the tDNA detection panel.

## Abbreviations

The following abbreviations are used in this manuscript:

BC	Bladder cancer
UBC	Urothelial bladder cancer
PCR	Polymerase chain reaction
ddPCR	Digital droplet polymerase chain reaction
tDNA	Tumor DNA
CHIP	Clonal hematopoiesis of indeterminate potential
MAF	Mutant allele fraction
*pTERT*	Promoter of the *TERT* gene
ROC	Receiver operating characteristic
AUC	Area under the curve
APOBEC	Apolipoprotein B mRNA-editing enzyme catalytic polypeptide-like
CI	Confidence interval
