# Supplementary material for: The Diagnostic Performance of a Four-Gene Digital Droplet PCR Panel for Urine Liquid Biopsy in Urothelial Bladder Cancer
Source: Diagnostics (Basel). 2025 Dec 24;16(1):69. doi: 10.3390/diagnostics16010069 (PMC12785868; doi:10.3390/diagnostics16010069)
Supplement: Supplementary file 1 [file diagnostics-16-00069-s001.zip › Figure S1.pdf]

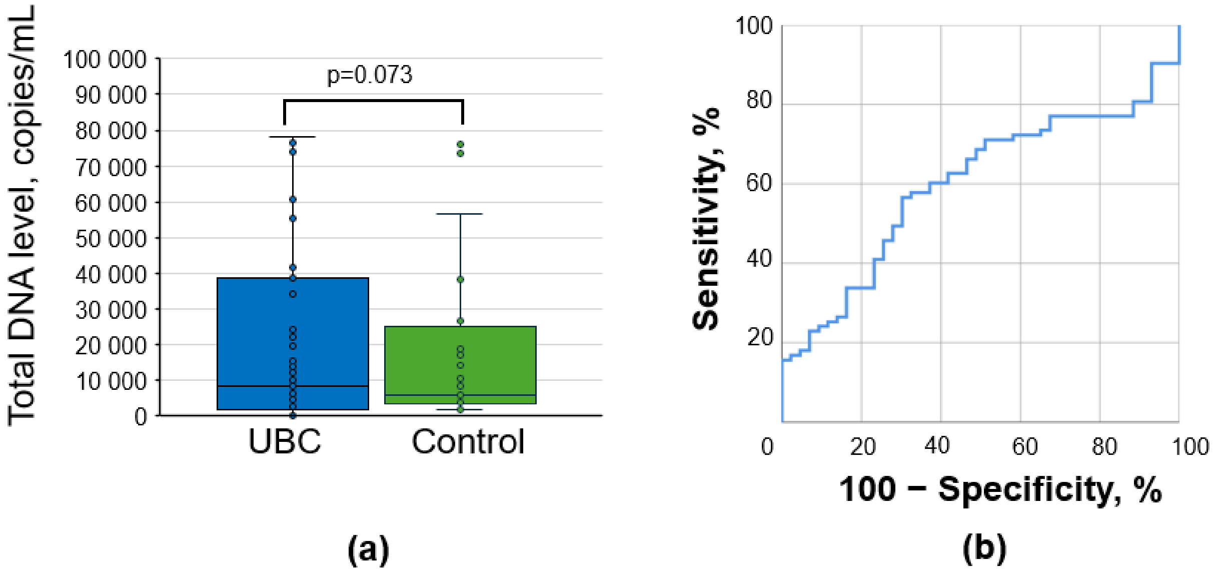

Figure S1. Comparison of total DNA levels in UBC and control groups. **(a)** Boxplots. **(b)** ROC-curves. Total DNA is presented as copies of human genome per 1 mL of urine (analyzed using ddPCR using the described assay for *pTERT* mutations analysis). Data were compared using Mann-Whitney U test.
