# Supplementary material for: The Diagnostic Performance of a Four-Gene Digital Droplet PCR Panel for Urine Liquid Biopsy in Urothelial Bladder Cancer
Source: Diagnostics (Basel). 2025 Dec 24;16(1):69. doi: 10.3390/diagnostics16010069 (PMC12785868; doi:10.3390/diagnostics16010069)
Supplement: Supplementary file 1 [file diagnostics-16-00069-s001.zip › Figure S3.pdf]

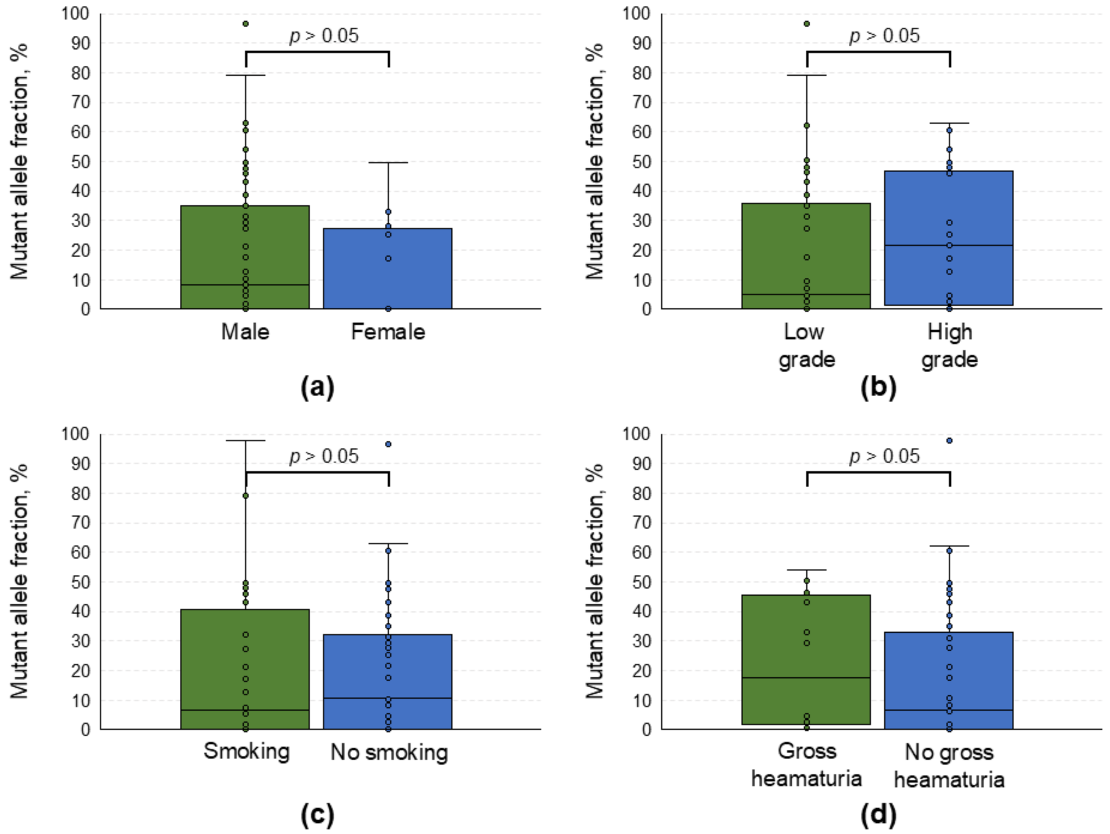

Figure S3. Mutant allele fractions in relation to various clinical and demographic parameters. **(a)** Sex. **(b)** Tumor grade. **(c)** History of smoking. **(d)** Macrohematuria. Mutant allele fractions were analyzed based on mutations in *pTERT, GPR126, FGFR3, PIK3CA*. *pTERT* mutations included substitutions in two positions upstream of the transcription starting site (at –124 bp, chr. 5: 1,295,228, G/A substitution; and –146 bp, chr. 5: 1,295,250, G/A substitution, genome assembly GRCh37). PIK3CA mutations included 3 most common substitutions in the coding regions of the gene (E545K, E542K, H1047R). *GPR126* mutations included two substitutions in its 6th intron (chr. 6: 142,706,206, G/A substitution, and chr. 6: 142,706,209, C/T substitution, genome assembly GRCh37). For *FGFR3* only 1 coding mutation was analyzed, namely S249C. If a sample was simultaneously positive for 2 or more mutations, mutant allele fraction was selected for a mutation with the highest value of the corresponding variable. *pTERT*, promoter of the *TERT* gene.
