# Supplementary material for: The Diagnostic Performance of a Four-Gene Digital Droplet PCR Panel for Urine Liquid Biopsy in Urothelial Bladder Cancer
Source: Diagnostics (Basel). 2025 Dec 24;16(1):69. doi: 10.3390/diagnostics16010069 (PMC12785868; doi:10.3390/diagnostics16010069)
Supplement: Supplementary file 1 [file diagnostics-16-00069-s001.zip › Figure S5.pdf]

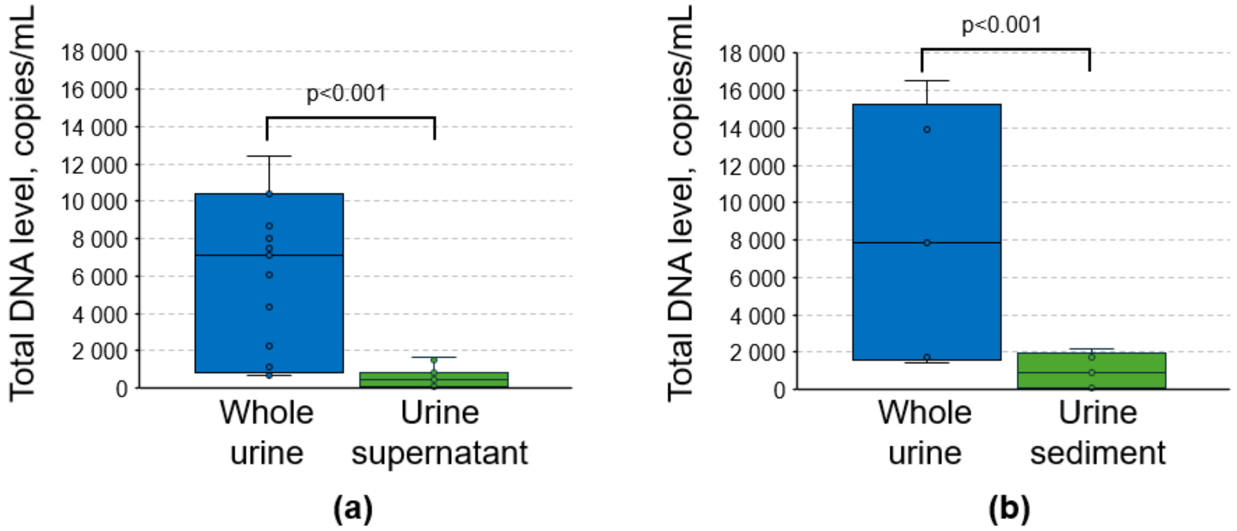

Figure S5. Comparison of total DNA levels for frozen biomaterial. **(a)** Whole urine vs. urine supernatant (n = 19). **(b)** Whole urine vs. urine sediment (n = 5). Total DNA is presented as copies of human genome per 1 mL of urine (analyzed using ddPCR using the described assay for *FGFR3* S249C mutation analysis). Whole urine samples (15 mL) were centrifuged at 2000 g for 15 min to separate urine sediment and supernatant. DNA was isolated from 4 mL of whole urine and urine supernatant using QIAamp Circulating Nucleic Acid kit (Qiagen GmbH, Hilden, Germany), and from 200 μL of urine sediment using QIAamp Mini kit (Qiagen GmbH, Hilden, Germany). Each pair of biomaterials was obtained from same individuals with UBC. Data were compared using Wilcoxon signed-rank test for paired variables. Data for the comparison of whole urine and urine sediment must be interpreted with caution due to following reasons. Firstly, the sample size for this comparison was quite limited. Secondly, in some cases the volume of sediment significantly exceeded the possible biomaterial input for DNA isolation. The excess of sediment was not accounted for during the calculation of total DNA level in copies/mL, as it would resemble multiple isolations of DNA from a sample, which is unlikely in a possible clinical scenario. Finally, DNA isolation was performed using different reagents due to differences in the structure and volume of the biomaterials.
